# Large scale germplasm screening for identification of novel rice blast resistance sources

**DOI:** 10.3389/fpls.2014.00505

**Published:** 2014-10-02

**Authors:** Kumar Vasudevan, Casiana M. Vera Cruz, Wilhelm Gruissem, Navreet K. Bhullar

**Affiliations:** ^1^Plant Biotechnology, Department of Biology, ETH Zurich (Swiss Federal Institute of Technology)Zurich, Switzerland; ^2^International Rice Research InstituteLos Banos, Philippines

**Keywords:** disease resistance, rice blast, rice germplasm, genetic diversity, blast resistance, *Pi2* locus

## Abstract

Rice is a major cereal crop that contributes significantly to global food security. Biotic stresses, including the rice blast fungus, cause severe yield losses that significantly impair rice production worldwide. The rapid genetic evolution of the fungus often overcomes the resistance conferred by major genes after a few years of intensive agricultural use. Therefore, resistance breeding requires continuous efforts of enriching the reservoir of resistance genes/alleles to effectively tackle the disease. Seed banks represent a rich stock of genetic diversity, however, they are still under-explored for identifying novel genes and/or their functional alleles. We conducted a large-scale screen for new rice blast resistance sources in 4246 geographically diverse rice accessions originating from 13 major rice-growing countries. The accessions were selected from a total collection of over 120,000 accessions based on their annotated rice blast resistance information in the International Rice Genebank. A two-step resistance screening protocol was used involving natural infection in a rice uniform blast nursery and subsequent artificial infections with five single rice blast isolates. The nursery-resistant accessions showed varied disease responses when infected with single isolates, suggesting the presence of diverse resistance genes/alleles in this accession collection. In addition, 289 accessions showed broad-spectrum resistance against all five single rice blast isolates. The selected resistant accessions were genotyped for the presence of the *Pi2* resistance gene, thereby identifying potential accessions for isolation of allelic variants of this blast resistance gene. Together, the accession collection with broad spectrum and isolate specific blast resistance represent the core material for isolation of previously unknown blast resistance genes and/or their allelic variants that can be deployed in rice breeding programs.

## Introduction

Crop plant diseases caused by various pathogens such as viruses, bacteria, oomycetes and fungi pose major challenges to global crop production and food security. Global climate change is predicted to further increase the negative impact of biotic stresses. Higher temperatures and erratic weather pattern are likely to change the geographical pathogen distribution. This in turn might decrease the effectiveness of existing resistance genes in crop varieties (Garrett et al., 2006; Milus et al., 2009) by promoting more aggressive races of pathogens. For example, more frequent wheat rust disease outbreaks, during the last years (2009, 2010, and 2013) threaten production in almost all wheat-growing countries and have caused heavy annual yield losses, sometimes as high as 50% in case of an early infection (Huerta-Espino et al., 2011; FAO, 2014). The recently emerged virulent stem rust (*Puccinia graminis*) race *Ug99* has overcome the major resistance gene *Sr31* (Pretorius et al., 2000), which is widely used in India, China, Europe and South America. Similarly, rice blast caused by the fungus *Magnaporthe oryzae* is one of the most devastating rice pathogens because it can infect the plant during nearly all growth stages. Repeated epidemics and frequent breakdown of rice blast resistance causing yield losses of 20–100% have been reported over the last decades in India and Japan (Khush and Jena, 2009; Sharma et al., 2012). Effective management of such pathogens require constant breeding efforts for development of resistant cultivars.

Plant defense involves various mechanisms that range from physical barriers such as the waxy cuticle, release of phytochemicals (e.g., phenols and terpenoids), quantitative resistance triggered by pathogen-associated molecular pattern (PAMP) recognition receptors, as well as various minor and major resistance genes acting against specific pathogen races (Yang et al., 2013). PAMP-mediated immunity is a baseline defense system in plants during which transmembrane receptors activate resistance upon detection of conserved pathogen-associated molecules such as flagellins, lipopolysaccharides and chitin that are shared by all bacterial and fungal pathogens (DeYoung and Innes, 2006; Jacob et al., 2013). Pathogens can evade this basal defense by releasing effector molecules, which often are recognized by intracellular resistance proteins encoded by major resistance (R) genes thereby providing effector-triggered plant immunity (ETI) (Jones and Dangl, 2006; Jacob et al., 2013). Major R genes encode five different classes of proteins (Dangl and Jones, 2001), with additional subclasses defined on the basis of domain structures as well as their membrane topology (Jacob et al., 2013). The nucleotide binding site and leucine rich repeats (NBS-LRR) type form the largest class of R proteins that have either a coiled coil (CC) or a TOLL/interleukin 1 receptor (TIR) domain at the N-terminus. Each of these domains have their own structural and functional significance, for example the LRR domain that contains highly conserved segments (HCS) as well as variable segments (VS) is known to be involved in protein-protein or protein-ligand interactions (Matsushima and Miyashita, 2012). Approximately 500 NBS-LRR genes have been identified in the rice genome (Monosi et al., 2004) and about 150 genes in the Arabidopsis genome (Yang et al., 2013). The R genes are being widely used in crop breeding for protection against various diseases.

Advancements in molecular techniques, whole genome sequencing and functional genomics have facilitated the identification, fine mapping and cloning of several R genes in the recent years. Information on molecular markers linked to these genes is increasingly available and used in marker-assisted breeding programs to provide effective and broad resistance in the field. Pyramiding of major R genes that vary in their mode of action and confer resistance against various pathogens has been successfully deployed (Datta et al., 2002; Maruthasalam et al., 2007). For example, pyramiding of *xa5*, *xa13*, and *Xa21* (Singh et al., 2001) has broadened resistance against bacterial blight (*Xanthomonas oryzae*) and of *Rsv1*, *Rsv3*, and *Rsv4* against soybean mosaic virus (Shi et al., 2009) as compared to varieties carrying single R genes only. Additionally, mixed planting of crop varieties with different *R* genes has been recommended as an alternative for effective utilization of host plant resistance. Mixing varieties or multilines with varying levels of resistance and susceptibility reduce the disease pressure and severity in the field compared to planting of single resistant varieties that are more vulnerable to disease outbreaks (Zhu et al., 2000; Raboin et al., 2012). The usefulness of this strategy has been shown over the last decades in experiments for the control of fungal diseases (Mundt, 2002; Castilla et al., 2010). For example, Zhu et al. (2000) demonstrated its effectiveness in China for the control of rice blast, where susceptible sticky rice was planted as single rows interspersed between four to six rows of resistant hybrid rice. This greatly reduced rice blast infection (>90%) and increased grain production (>85%) in the mixture plots as compared to the monocultures. However, not all or random mixtures combinations will be functional because several controlling factors, including epidemiological conditions and varied degrees of susceptibility to different pathogens, can affect outcome. Therefore, the multi-variety strategy requires adaption to local agricultural priorities and available resistant genotypes. For example, in the uplands of Indonesia, farmers are willing to adopt variety mixture if these varieties have similar height, growth duration and grain quality (Vera Cruz et al., 2009).

Most plant pathogens are fast evolving and can break down the resistance conferred by R genes, thus resulting in disease epidemics. These can be influenced by several factors, including weather conditions, disease pressure, as well as genome stability of the pathogen. The *M. oryzae* genome is rich in repetitive segments and retro-transposons (Dean et al., 2005), which allow the fungus to frequently change pathogenicity or escape from host recognition by altering the effector molecules. This requires the continuous identification of new sources of host disease resistance against continuously evolving and geographically diverse pathogen races. Crop germplasm collections maintained in genebanks at centers such as the International Rice Research Institute (IRRI) in the Philippines, the Leibniz Institute of Plant Genetics and Crop Plant Research in Germany, the International Maize and Wheat Improvement Center (CIMMYT) in Mexico, the International Potato Center (CIP) in Peru, Bioversity International in Belgium, or the Svalbard Global Seed Vault in Norway provide access to repertoires of favorable genes. These germplasm collections include several traditional cultivars and landraces as well as wild species from various geographical origins. The landraces and wild relatives may not possess favorable agronomic traits (such as plant height or yield) suitable for modern breeding programs, but they are highly relevant sources of genes for specific traits, including resistance or tolerance to biotic and abiotic stresses. Resistance to late blight (*Phytophthora infestans*) in potato derived from diverse wild *Solanum* species (Vossen et al., 2003; Foster et al., 2009), identification of broad spectrum rice blast resistance genes such as *Pi9* from *Oryza minuta* (Qu et al., 2006) and the phosphorus uptake (*Pup1*) locus in rice (Gamuyao et al., 2012) from the traditional cultivar Kasalath are convincing examples of important contributions of wild species and traditional cultivars to our breeding programs.

Here we report the evaluation of 4246 rice gene bank accessions to identify new rice blast resistance sources. We selected rice accessions of diverse geographic origins on the basis of their annotated rice blast resistance in the IRRI rice germplasm collection. Systematic screening for rice blast resistance was carried out under both field and controlled environmental conditions. Using this approach we identified candidate accessions that can be deployed as donors in rice breeding programs and as starting material for the isolation of novel rice blast resistance genes and allelic variants of major rice blast resistance genes.

## Materials and methods

### Plant materials

The rice germplasm material was obtained from the International Rice Genebank (IRG) of the International Rice Research Institute (IRRI), Philippines. The International Rice Germplasm Collection (IRGC) of IRG holds more than 120,000 accessions from different geographical regions of the world (129 countries). The accessions for our study were selected on the basis of documented rice blast resistance information i.e., characterized as resistant, moderately resistant or susceptible, in the International Rice Germplasm Collection Information System (IRGCIS) of IRG. The rice cultivars IR72 and CO39 were used as susceptible control lines throughout the study. Blast monogenic resistant lines for *Pi2* (IRBLz5-CA), *Pi9* (IRBL9-W), *Piz-t* (IRBLzt-T), *Pita* (IRBLta-CP1), *Pi54* (IRBLkh-K3), and *Pib* (IRBLb-B) were used as control lines for isolate specific rice blast resistance evaluation.

### Magnaporthe oryzae isolates

For primary screening in the Uniform Blast Nursery (UBN), a mixture of naturally existing blast strains in the IRRI locality was used. Diseased plantlets of blast susceptible lines IR72 and CO39 were taken from IRRI pathology fields and were used as a source of inoculum for spreading the disease on control/disease-check lines in nursery screening beds.

For the second level screening under greenhouse conditions, the five highly virulent *M. oryzae* isolates (Supplementary Figure 1) M101-1-2-9-1, M39-1-2-21-2, JMB8401, M64-1-3-9-1, and Ca41 were selected based on their reported differential disease pattern/spectrum on blast monogenic lines carrying blast resistant genes *Pi2*, *Pi9*, *Piz-t*, *Pi54*, *Pib*, and *Pita* (Kobayashi et al., 2007; Telebanco-Yanoria et al., 2008). Protocols reported by Hayashi et al. (2009) were followed for the maintenance, culture and sporulation of individual rice blast isolates.

### Inoculation and disease scoring

For UBN screening, test accessions (30 plants/test entry) were sown on nursery beds, along with a mixture of varieties with differing level of resistance to local *M. oryzae* strains sown as borderlines/spreader rows. Additionally the susceptible controls were sown as one line of IR72 for every 10 lines of test accessions and one line of CO39 for every 100 lines of test accessions. Inoculation was done on border lines (as described above) after 10 days from the date of sowing and disease was allowed to spread naturally via wind dispersal. Nursery beds were water sprayed 3–4 times per day and were covered during the night to maintain a high humidity until disease development and progression was observed in border lines.

In case of greenhouse screening, test accessions (15 plants/test entry) were sown in plastic trays (10 rows × 2 columns per tray) in five batches for inoculation with five individual blast isolates mentioned above. Plants were inoculated 10 days after sowing using 50 ml of spore suspension solution with a concentration of 1 × 10^5^ spores per ml with 1% tween-20 per tray. After inoculation, the plants were maintained in moist chamber at 26–28°C for 24 h and after this period, the plants were transferred to the incubation chamber at 25°C ± 2 for 1 week before disease assessment.

Disease evaluations were performed for each test line according to the Standard Evaluation System of IRRI (1996), for screening leaf blast, with scores ranging from 0 to 9 (Supplementary Figure 2). Blast lesion type and the leaf area covered with infection were recorded. In this scoring system, score 0 represents no blast lesions observed and is graded as highly resistant, whereas score 9 marks as highly susceptible with more than 75% leaf area infected. The phenotypic scores of 0, 1, 2, and 3 are considered as blast resistant in the rice breeding programs and data were collected accordingly in the screening experiments.

### Molecular characterization of candidate accessions

Leaf samples were collected from the plants resistant to rice blast (scale 0–3) in UBN for genomic DNA isolation. Ground leaf samples were collected in 2 ml microcentrifuge tubes, 1.2 ml of 2 × CTAB-buffer was added, and the samples were incubated at 65°C for 30 min. After addition of 800 μl of phenol:chloroform:isoamylalcohol (25:24:1) and mixing, the sample tubes were centrifuged for 15 min at 13,000 rpm. The aqueous supernatant was collected in new 1.5 ml tubes and was treated with RNaseA at 37°C for 30 min. DNA was precipitated by adding 560 μl of isopropanol and pelleted by centrifugation at 13,000 rpm for 10 min. After washing twice with ethanol, the dried DNA pellet was dissolved in 50 μl of 1 × TE buffer and was used for polymerase chain reaction (PCR) analysis.

PCR was carried out to determine the presence/absence of *Pi2* locus using a Sequence Tagged Site (STS) marker (as reported in Zhou et al., 2006) that co-segregates with the *Pi2* gene. Forward primer: 5′-TCTATAGAAGTGCAAACAGC and Reverse primer: 5′-TTAGGTACGAACATGAGTAG were used to amplify the targeted fragment of ~2 kb from the test accessions. The PCR program included initial denaturation at 95°C for 5 min; followed by additional denaturation at 95°C for 30 s, annealing at 51°C for 30 s, extension at 72°C for 1 min (these three steps repeated for 39 cycles); followed by final extension at 72°C for 7 min. Genomic DNA extracted from *Pi2* monogenic line was used as the positive control and that from susceptible rice cultivar LTH was used as the negative control, in addition to a water control. In addition, rice actin gene was amplified within the same sample serving as an internal PCR control (actin forward primer: 5′-TTATGGTTGGGATGGGACA; actin reverse primer: 5′-AGCACGGCTTGAATAGCG).

## Results

### Selection of diverse rice germplasm from IRGCIS database

The IRGCIS online database contains 6338 rice accessions that are annotated as resistant to rice blast (excluding moderately resistant accessions) within the total rice germplasm collection at IRG. From these 6338 accessions, we selected genotypes of known varietal groups originating from 13 major rice growing countries, providing a total of 4246 rice genotypes as the starting material (excluding 96 accessions for which no seed was available or there was no germination). The 13 countries (and number of rice genotypes) chosen for this selection were India (1457), Indonesia (775), Bangladesh (613), Thailand (355), Philippines (333), Vietnam (248), China (116), Myanmar (92), Taiwan (76), Brazil (68), Japan (59), Nepal (47), and South Korea (7). These represent major rice producing and consuming countries. The accessions were assigned to three major varietal groups, i.e., indica (85.9%), javanica (9.2%), and japonica (4.9%). The 13 countries cover 66.9% of the annotated blast resistant genotypes within the full rice germplasm collection available at IRG while the remaining 33.1% is distributed over a total of 55 countries.

### Screening of candidate accessions for rice blast resistance in the field condition

**Figure 1:**
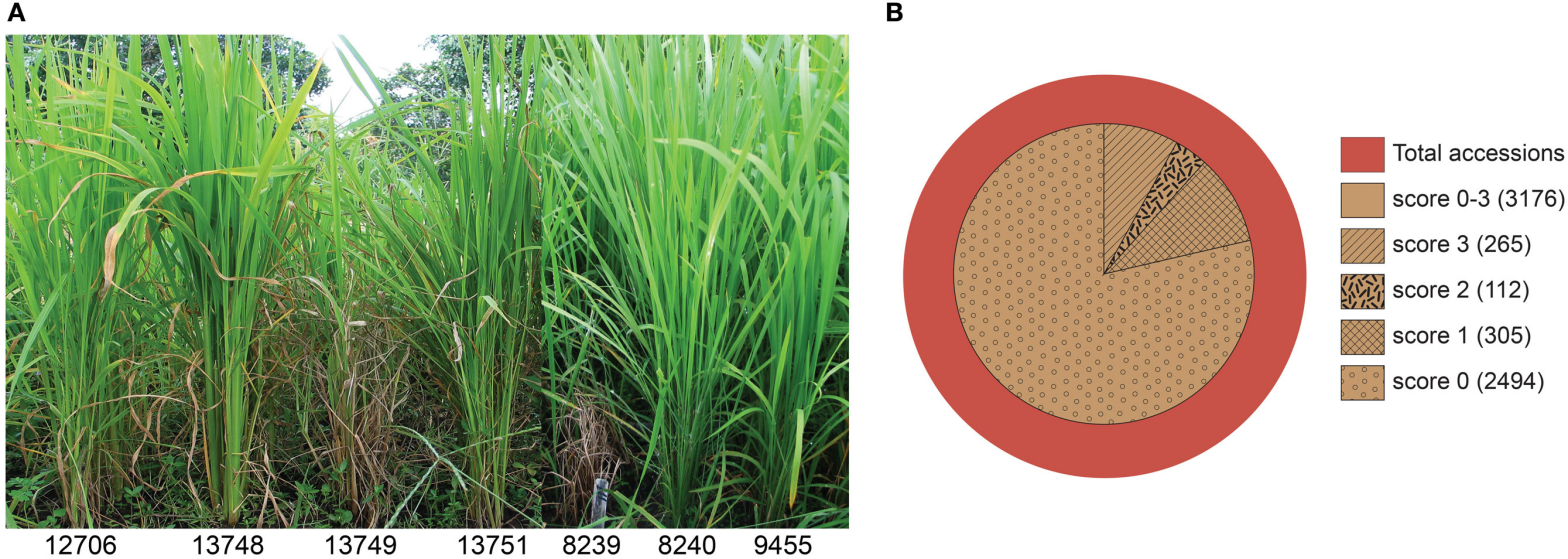
**Rice blast resistance screening in the uniform blast nursery. (A)** Sample images of rice genotypes showing full blast resistance phenotypes (IRGC 12706, 13748, 13751, 8240, and 9455) in contrast to susceptible genotypes (IRGC 13749 and 8239) in the uniform blast nursery. **(B)** Summary of rice blast resistance screening in the uniform blast nursery. Inner circle represent the number of accessions that scored as resistant (score 0–3) using the 0–9 scoring system. Majority of the accessions were observed with score 0 (78.5%) while accessions with score 1, 2, and 3 accounted for 9.6, 3.5, and 8.34% of the 3176 resistant accessions, respectively. The numbers of resistant accessions in each respective category are shown in brackets.

The 4246 selected rice accessions were screened for blast resistance in the uniform blast nursery (Figure 1). A large majority of the rice genotypes exhibited complete resistance when compared to the susceptible control lines. We found 3176 (74.8%) accessions to be blast resistant with scores between 0 and 3 (Figure 1 and Table 1). Most of these accessions scored as highly resistant with score 0, accounting for 78.5% of the 3176 resistant genotypes and 58.7% of the total 4246 genotypes screened. Although each country had a high number of resistant accessions, the percentages of blast resistant genotypes from different countries varied (Table 1), with Brazil containing the highest number of resistant accessions (95.6% of Brazilian accessions screened) while Japan had the lowest number (54.2% of Japanese accessions screened). For the other 11 countries, the percentage of resistant accessions ranged between 57.1 and 89.6% based on the number of genotypes screened from the respective country (South Korea 57.1, China 60.3, Bangladesh 63.1, India 70, Nepal 70.2, Vietnam 75, Philippines 75.9, Myanmar 80.4, Taiwan 82.9, Indonesia 86.6, and Thailand 89.6%). Within these resistant genotypes, 2726 (85.8%), 295 (9.2%), and 155 (4.9%) belong to indica, javanica and japonica varietal groups, respectively. Additionally, a relatively smaller set of 588 accessions exhibited moderate resistance with scores 4 and 5 (Supplementary Figure 3). The moderately resistant accessions were identified in all the 13 countries, with India, Bangladesh and Indonesia being the major contributors covering 75.5% of the total moderately resistant accessions.

**Table 1:** **Summary of rice genotypes screened as resistant in uniform blast nursery**.

**Country of origin**	**Number of genotypes screened**	**Score 0–3**	**Score 0**	**Score 1**	**Score 2**	**Score 3**
India	1457	1020	934	43	16	27
Indonesia	775	671	525	54	24	68
Bangladesh	613	387	292	27	17	51
Thailand	355	318	216	74	12	16
Philippines	333	253	210	13	9	21
Vietnam	248	186	97	40	16	33
China	116	70	44	14	4	8
Myanmar	92	74	42	16	2	14
Taiwan	76	63	54	3	0	6
Brazil	68	65	56	5	1	3
Japan	59	32	7	3	8	14
Nepal	47	33	14	12	3	4
South Korea	7	4	3	1	0	0

The number of accessions found resistant in the uniform blast nursery for each country is presented. Number of genotypes is further detailed for disease evaluation scores 0, 1, 2, and 3.

**Figure 2:**
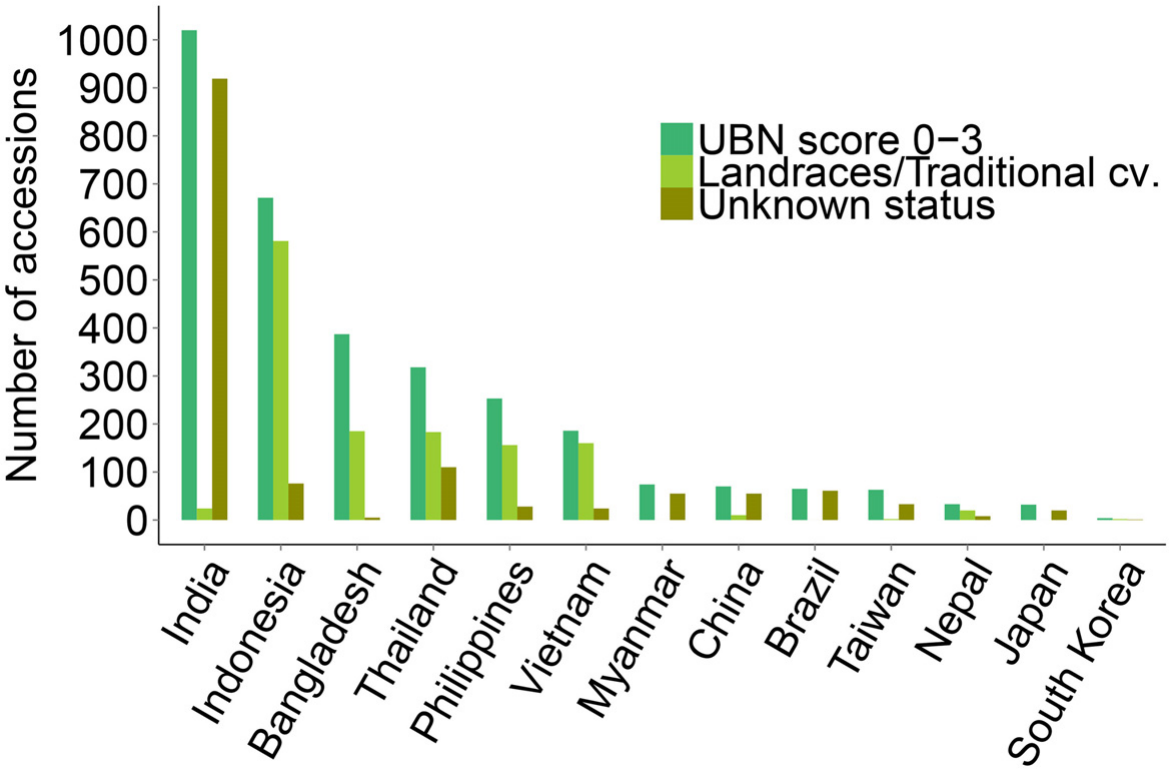
**Landraces/traditional cultivars among identified resistant rice genotypes**. Landraces/traditional cultivars among the blast resistant genotypes from the respective countries are shown, together with the resistant genotypes of unknown status. India contributes nearly one third of the total blast resistant genotypes but 90% of the accessions have an unknown sample status. Excluding India, 60% of the blast resistant rice genotypes identified in the 12 remaining countries are traditional cultivars/landraces. None of the resistant accessions from Myanmar, Brazil and Japan were landraces/traditional cultivars but a large numbers of identified resistant cultivars from these countries have an unknown status as well. A minor proportion of the UBN-resistant genotypes were inbred lines and improved varieties (data not shown).

Out of the total 3176 resistant accessions (score 0–3), 1323 accessions (41.6% of total resistant) are annotated as traditional cultivars or landraces in the IRGCIS database (Figure 2), demonstrating the potential of landraces as promising sources of novel resistances. Considering that not all of the accessions in the database are fully annotated, it is likely that the number of resistant landraces is even higher. For example, in the case of India that contributes nearly one third (1457 accessions) of our study material, 88.7% of the accessions are annotated as “unknown sample status.” For the other 12 countries the number of resistant accessions with traditional cultivars/landraces status is 60% of the total blast resistant accessions. It should be noted, however that these countries also contain samples of unknown status (Figure 2).

### Isolate specific screening of the UBN resistant accessions

**Figure 3:**
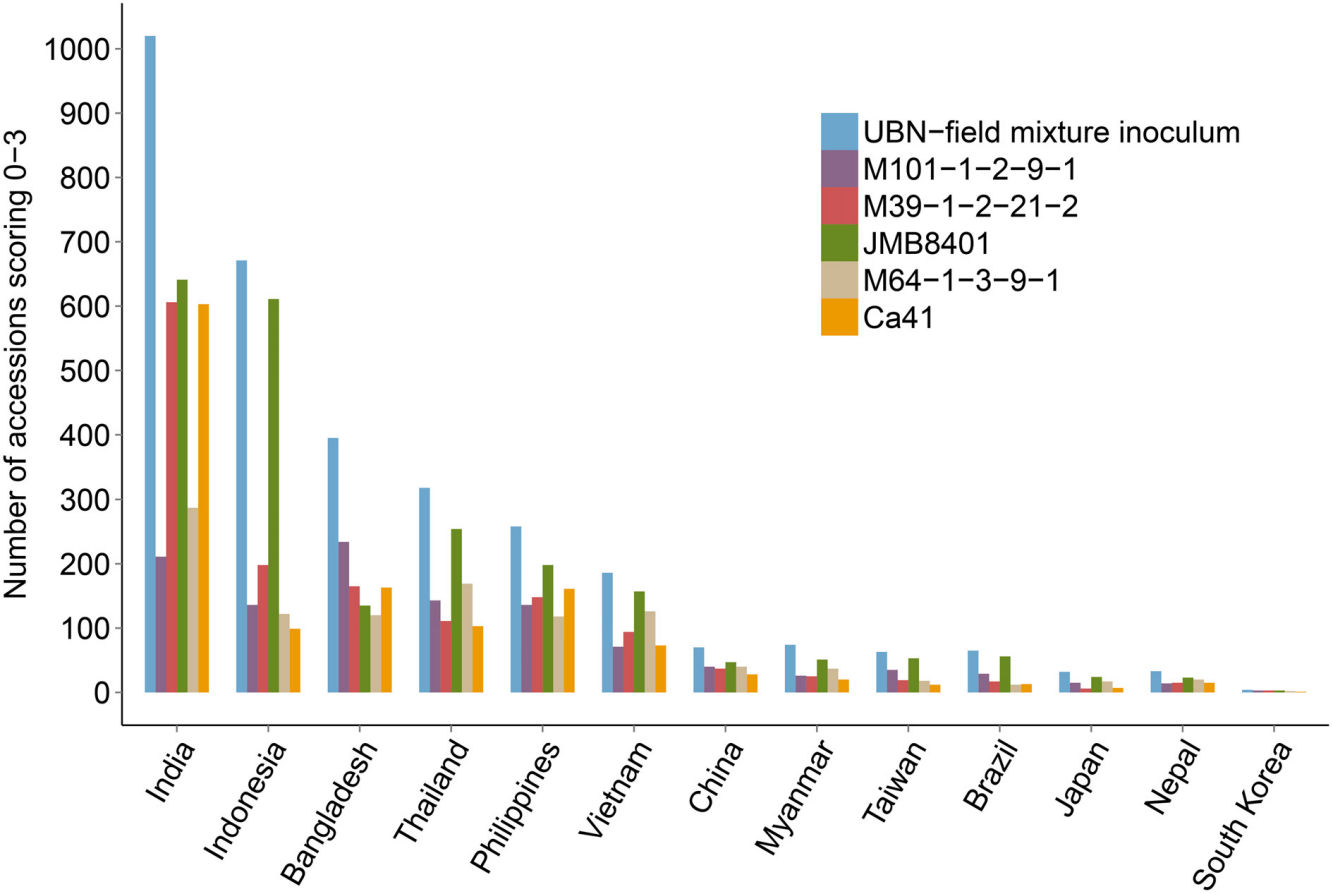
**Isolate specific screening of UBN resistant rice genotypes**. UBN-resistant accessions (score 0–3) were screened against five different single rice blast isolates under controlled conditions. Data for accessions exhibiting resistance against particular isolates is shown, with different color bars representing each isolate. It should be noted that several accessions exhibited resistance against two or more isolates. The screened rice accessions displayed varied patterns of disease symptoms, with accessions resistant or susceptible only to one blast isolate as well as accessions that exhibited resistance against two to all of the isolates.

The rice genotypes that were found to be resistant to rice blast (score 0, 1, 2, and 3) in the UBN screening were subjected to isolate-specific screening using five different *M. oryzae* isolates (M101-1-2-9-1, M39-1-2-21-2, JMB8401, M64-1-3-9-1, and Ca41). The isolates exhibit varied disease pattern, either unique or shared between different monogenic lines (see Materials and Methods). We expected that using these isolates for screening would facilitate the identification of potentially novel and broad-spectrum rice blast resistance sources. Out of the total 3176 UBN-resistant rice accessions, the genotypes resistant against the individual blast isolates (score 0–3) were 1093 for M101-1-2-9-1, 1444 for M39-1-2-21-2, 2253 for JMB8401, 1088 for M64-1-3-9-1, and 1298 for Ca41 (Figure 3). As expected, a varied degree of disease susceptibilities were observed among different rice genotypes when scoring against different *M. oryzae* isolates (Supplementary Figure 4, Supplementary Figure 5). For example, the variety DV109 (IRGC Nr. 8854) was highly resistant in the UBN screening and against the isolate M101-1-2-9-1, moderately resistant against Ca41, moderately susceptible against M39-1-2-21-2, and JMB8401 while it scored highly susceptible against M64-1-3-9-1. Such resistance and susceptibility pattern against different isolates identify these accessions as potential novel sources of race-specific resistance genes.

We found a small set of 262 genotypes to be susceptible to all five *M. oryzae* isolates. However, these accessions might have resistance against isolates not included in our current study. It is also possible that these accessions have partial resistance but could become susceptible against a specific highly virulent isolate.

### Identification of genotypes conferring broad-spectrum resistance

**Figure 4:**
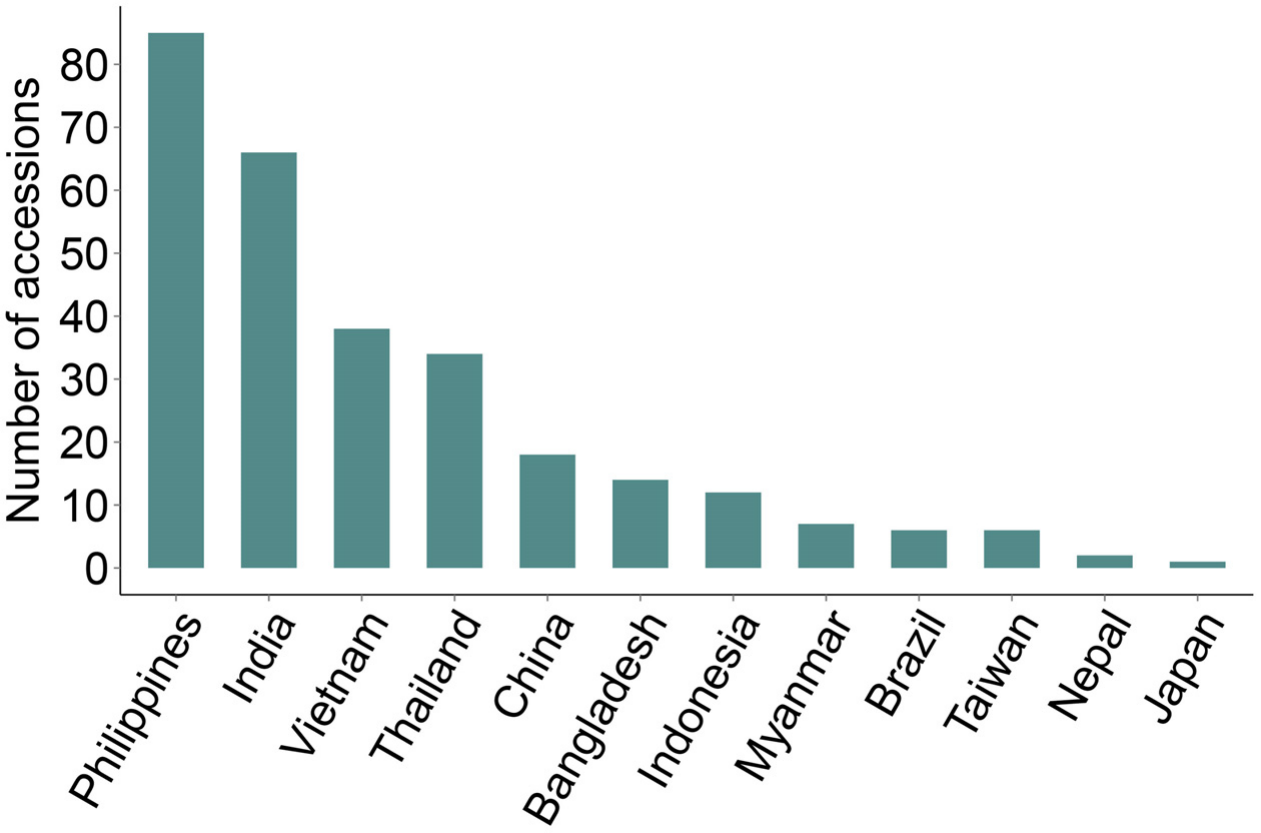
**Broad spectrum resistant rice genotypes**. Rice accessions showing broad spectrum UBN-resistance against the field mix inoculum as well as against all the five individual rice blast isolates are presented. 289 broad-spectrum resistance rice accessions were identified (scores 0–3), with Philippines, India, Vietnam, Thailand and China being the major contributors. None of accessions from South Korea was identified as broad spectrum resistant.

We identified 289 genotypes that showed broad-spectrum resistance, of which 39.4% are landraces. These genotypes were found resistant (scale 0–3) to blast, both in the uniform blast nursery and when inoculated with the five individual rice blast isolates (Figure 4, Supplementary Table 1). The Philippines, India, Vietnam and Thailand contributed more broad-spectrum blast resistant genotypes (29.4, 22.8, 13.1, and 11.8%, respectively) among the 289 genotypes identified as broad-spectrum resistant (Figure 4). Nearly 30% of the 289 broad-spectrum resistant genotypes originated from the Philippines, which is high proportion considering that only 7.8% (333 accessions) of the total 4246 accessions screened were from Philippines (Table 1). This is expected because the rice accessions were challenged with *M. oryzae* isolates from the Philippines and these accessions likely have specific resistances against local isolates. Nevertheless, these accessions are promising donors for breeding local rice varieties in the Philippines and in other rice growing agricultural systems across Asia. It is also likely that these broad spectrum genotypes have multiple functional blast resistance genes and/or QTLs, which could be useful for detailed molecular analysis to identify the underlying blast resistance genes.

### Molecular screening for identification of accessions carrying *Pi2* rice blast resistance locus

**Figure 5:**
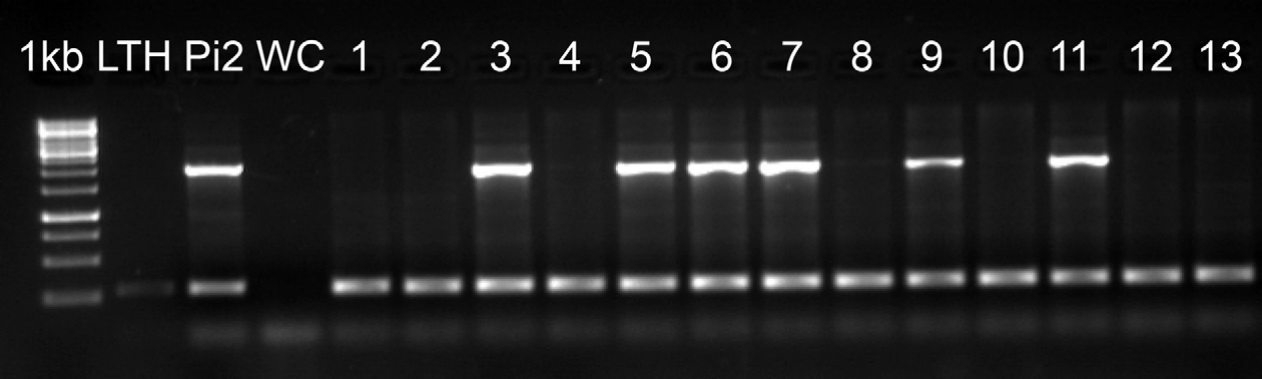
**Molecular screening of UBN resistant genotypes for *Pi2* locus**. Molecular screening of 3176 UBN-resistant rice genotypes revealed 794 genotypes carrying the *Pi2* locus. 1 kb, DNA marker; LTH, negative control; *Pi2*, positive control (*Pi2*-monogenic line); WC, water control; 1–13, test accessions. The accessions positive for *Pi2* are 3 (IRGC 254), 5 (IRGC 257), 6 (IRGC 258), 7 (IRGC 840), 9 (IRGC 846), and 11 (IRGC 877). The upper band (~2 kb) indicates the presence of *Pi2* locus and the lower band (~250 bp) shows the actin band, which is an internal control for the PCR.

We screened the rice accessions that were blast resistant in the UBN screen for the *Pi2* locus using a STS marker (Zhou et al., 2006). The *Pi2* locus represents a complex cluster of multiple NBS-LRR genes with different resistance specificities. Three genes (*Pi2*, *Pi9*, and *Piz-t*) have been cloned and another four genes (*Pi40(t)*, *Pigm*, *Pi26*, and *Piz*) have been mapped to this locus. All of them confer broad spectrum resistance against rice blast, but at the same time these genes vary in their resistance pattern against different *M. oryzae* isolates (Qu et al., 2006; Liu et al., 2010; Wu et al., 2012). Among the 3176 rice accessions that were blast resistant in UBN screen, we identified 794 genotypes that carry the *Pi2* locus (Figure 5, Supplementary Table 2). It is interesting to note that half of the 289 broad-spectrum blast resistant genotypes (50.2%) carry the *Pi2* locus. These resistant accessions found to carry *Pi2* locus are promising lines for the identification of allelic variants of the *Pi2/Pi9/Piz-t* genes and as well for the identification and cloning of other broad-spectrum resistant genes that were mapped to this locus.

## Discussion

### Identification of genetic variation is the key to future crop improvement

Domestication and modern breeding has reduced genetic diversity of crop plants (Tanksley and McCouch, 1997) by replacing landraces and traditional farmer cultivars with modern, high yielding varieties. Although this has allowed filtering out genes that cause detrimental traits, the reduction in genetic variation now limits the options of plant breeders to develop new varieties with the existing germplasm. New varieties are constantly needed to meet consumer demands and for protection of crops against highly unpredictable biotic and abiotic stresses that are encountered in agricultural systems. Breeding of improved varieties requires the identification of novel functional genes or alleles, and this calls for making effective use of our global crop genetic resources available in seed bank collections. These seed collections represent a wide range of genetic diversity that is critical for maintaining and enhancing the yield potential and other quality traits, because they can provide new sources of resistance and tolerance to various stresses. Many of the genes for highly relevant traits in modern crop cultivars have been transferred from their wild relatives and landraces, for example the *Rht* and *sd-1* genes that confer dwarf stature in rice and wheat, respectively (Hedden, 2003; Hoisington et al., 1999). In the case of rice, the six wild species *O. rufipogon*, *O. longistaminata*, *O. nivara*, *O. breviligulata*, *O. glumaepatula*, and *O. meridionalis* together with the two cultivated species *O. sativa* and *O. glaberrima* have been identified as the primary gene pool for rice cultivars because genes can be easily transferred between these species (Khush, 1997). The wild species have been used as a source of many relevant genes for rice breeding, and *O. rufipogon* has been suggested as source of broad-spectrum rice blast resistance genes (Ram et al., 2007). Other examples include the bacterial blight resistance gene *Xa21* that was first identified in *O. longistaminata*, the broad-spectrum blast resistance gene *Pi-hk1* that was identified in the landrace Heikezijing (Wu et al., 2013), and the submergence tolerance gene *SUB1* from the landrace FR13A (Bailey-Serres et al., 2010). Furthermore, *Pi-Da(t)* identified in an upland rice variety Dacca6 in Jin23B background has been used in an elite parental line (Shi et al., 2012), not only for its blast resistance but also for its good grain quality as well as for wide adoption for three-line hybrid rice breeding program over the past 20 years in China. In addition to traits for biotic and abiotic stress resistance or tolerance, rice landraces also served as an important source for yield traits such as *SPIKE* (an allele of *NARROW LEAF1*; *NAL1*) from an Indonesian rice landrace that increased rice grain yield by 18% (Fujita et al., 2013) and *TGW6* (*THOUSAND-GRAIN WEIGHT 6*) from the Indian landrace Kasalath, which significantly increases grain yield as well (Ishimaru et al., 2013).

Despite the high genetic potential and efforts of identifying diversity from germplasm collections, the genetic base of our major crops remains narrow. Adaptation of pathogens and susceptibility to other stresses are continuous threats to existing elite crop varieties. Although there are demonstrated and valuable contributions of crop diversity to counter these threats, there is still a great potential hidden in available landraces, cultivars and wild species that remains under-explored. Large numbers of probably redundantly stored gene bank accessions and missing genotype × phenotype information make it difficult for modern breeding programs to select a feasible number of accessions for scoring traits of interest. Core collections have therefore been promoted as a means of exploiting large seed collections using smaller subsets of accessions that represent maximum diversity. However, while core collections aim to maximize genetic diversity in general, plant breeders and/or biologists are often interested in one or a few specific traits at a time. Other approaches such as the “Focused Identification of Germplasm Strategy (FIGS)” were therefore suggested to utilize eco-geographical information for identifying sets of gene bank accessions that are expected to have a maximum of functional diversity for one specific trait of interest (Bhullar et al., 2009).

We focused on potential rice accessions as donors of novel blast resistance alleles by selecting a test set based on annotated rice blast resistance information available in the IRGCIS database. Rice blast remains one of the most destructive diseases of rice, and resistance gene deployment has been suggested as most effective and environment friendly way of managing the disease. However, the continuously evolving genome of *M. oryzae* as well as existence of geographically diverse strains are challenging for the rice breeders. Genome studies of the rice blast fungus has revealed high probabilities of transposons mediated inactivation of genes involved in host specificity. Moreover, the high genetic variability in *M. oryzae* allows the fungus to broaden the host range and infect formerly resistant genotypes (Dean et al., 2005). It is therefore important to build a repertoire of resistant accessions/donors that could be screened and deployed in breeding programs according to needs of local agricultural systems. The rice accessions we selected from 13 major rice growing countries represent a broad geographic diversity. Nearly 75% of the UBN-screened accessions exhibited resistance to the local field mixture inoculum in the Philippines. We observed varied resistance patterns among these accessions when infected with five individual rice blast isolates. The severity of host plant disease phenotypes depends on interactions between various plant R genes or their allelic variants and corresponding pathogen effector molecules (DeYoung and Innes, 2006). Thus, the patterns of disease reactions against five differential isolates of rice blast varying from highly resistant to highly susceptible that we observed in these accessions indicate the presence of race specific genes/alleles and/or their combinations. Furthermore, 289 accessions conferred broad-spectrum resistance because they were resistant against the field-mixture inoculum as well as all the five tested blast isolates. Besides these completely resistant accessions, we also identified accessions conferring moderate resistance against rice blast. Considering the apparent lack of durability of major gene-conferred disease resistance, lines conferring such partial resistance could serve as potential donors for identifying and characterizing weaker yet more durable sources of resistance. Together, the identified resistant and moderately resistant accessions represent a rich stock of starting material that can be used directly as donor parents in breeding programs, as well as for identification/isolation of race specific, broad-spectrum resistance and/or durable resistance sources against rice blast. Further, there are evidences of quantitative trait loci (QTLs) conferring resistance to multiple pathogens. For example, the wheat leaf rust QTL *Lr34* confers broad-spectrum and durable resistance to leaf rust, stripe rust and powdery mildew (Krattinger et al., 2009; Kou and Wang, 2010). Therefore, it would be highly interesting to test the lines showing broad-spectrum rice blast resistance in our study for their response to other economically important rice pathogens such as *Xanthomonas oryzae* pv. *oryzae* and *Rhizoctonia solani*.

### Approaches for identification of high value genes from crop germplasm

Systematic evaluation of germplasm collections for agronomically relevant traits is necessary to facilitate strategies for efficient utilization of crop diversity. Conventional as well as high-throughput methods can be used to tap this diversity, including molecular marker screening, allele mining, genome wide association studies (GWAS), single nucleotide polymorphism (SNP) genotyping, as well as eco-TILLING (a variation of the “Targeting Induced Local Lesions In Genomes” method to identify natural mutations). Genome wide association studies help to discover and dissect functional variation among diverse genotypes for useful but complex traits such as grain yield (Huang et al., 2012). Allele mining and SNP genotyping could be particularly rewarding for (but not limited to) resistance traits (Bhullar et al., 2009, 2010; Wang et al., 2010) where even a small allelic variation can cause major changes in gene function (e.g., resistance vs. susceptibility) or in resistance spectrum (Bryan et al., 2000; Zhou et al., 2006). Large efforts are also being made to sequence genomes of diverse rice accessions. Using sequencing-by-synthesis technology for more than 500 Chinese rice landraces allowed the successful construction of a high-density haplotype map of the rice genome and GWAS for 14 agronomic traits in the population of *O. sativa indica* subspecies (Huang et al., 2010). Similarly, molecular analysis of blast resistant accessions identified in this study hold great potential for the characterization and isolation of novel rice blast resistance genes. Novel allelic forms of major R genes could be readily identified and cloned from this set using gene specific markers combined with PCR-based amplification and sequencing approaches. We found that 794 of the 3176 accessions that we screened and 50% of the 289 broad-spectrum resistance accessions carry *Pi2/Pi2*-like resistance genes. *Pi2* shares high sequence homology with its other gene family members, including the cloned *Pi9* and *Piz-t* broad spectrum resistance genes. It is therefore likely that these accessions not only contain allelic variants of *Pi2* but also of other members within the gene cluster on chromosome 6 (Zhou and Wang, 2009; Wu et al., 2012). The accessions carrying *Pi2* that we identified are the best candidates for isolating allelic variants and possibly characterizing additional broad-spectrum resistance genes from this locus. As reported here for *Pi2*, the approach we have used can be similarly extended to any major rice blast resistance gene of interest.

Together, increasing genome information and identification of candidate germplasm for novel traits represents an unprecedented resource for future rice breeding programs. Information gained from the systematic and standardized characterization of gene bank accessions using modern molecular tools must also be integrated into databases. Proper cataloging of genome and phenotypic information will enable breeders and plant biologists to use the existing genotypic diversity in seed banks for evaluating the biological significance of molecular and phenotypic diversity.

## Author contributions

Navreet K. Bhullar, Casiana M. Vera Cruz, Wilhelm Gruissem and Kumar Vasudevan designed the screen, Kumar Vasudevan carried out the experiments, Kumar Vasudevan and Navreet K. Bhullar analyzed the data, Kumar Vasudevan and Navreet K. Bhullar wrote the manuscript, Navreet K. Bhullar, Wilhelm Gruissem and Casiana M. Vera Cruz edited the manuscript. All authors have read the manuscript and agree with its content.

### Conflict of interest statement

The authors declare that the research was conducted in the absence of any commercial or financial relationships that could be construed as a potential conflict of interest.
