# Supplementary material for: Large scale germplasm screening for identification of novel rice blast resistance sources
Source: Front Plant Sci. 2014 Oct 2;5:505. doi: 10.3389/fpls.2014.00505 (PMC4183131; doi:10.3389/fpls.2014.00505)
Supplement: Supplementary file 3 [file Image1.PDF]

Supplementary figures

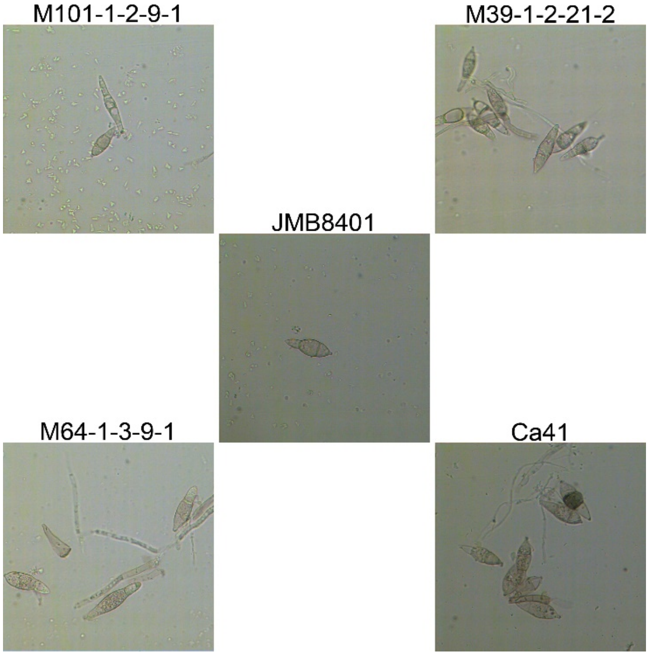

Supplementary figure 1. Individual rice blast isolates used in the study
Five rice blast isolates, namely, M101-1-2-9-1, M39-1-2-21-2, JMB8401, M64-1-3-9-1 and Ca41, were selected on the basis of their diverse pathogenicity patterns on rice monogenic lines with different major rice blast R genes. Conidiospores of these five isolates, as viewed under light microscope (40X), are presented.

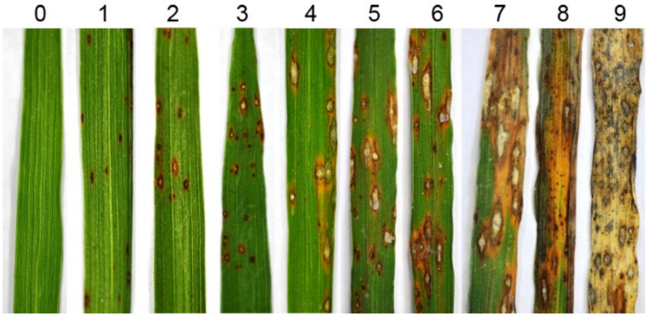

Supplementary figure 2. Standard evaluation scale with scores 0-9 for leaf blast.
Disease evaluation was done based on the lesion type observed, together with the leaf area covered with infection. For example, score 0 represents no disease observed/highly resistant and score 9 represents severe leaf necrosis/highly susceptible, while scores 1 to 8 mark gradual increase in the disease severity. Genotypes scoring 0, 1, 2 and 3 were considered as rice blast resistant and were selected for further evaluations.

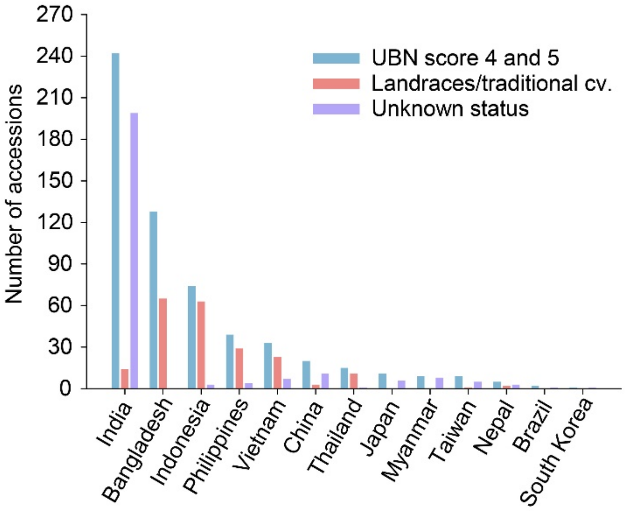

Supplementary figure 3.
Accessions exhibiting moderate resistance (scores 4 and 5)
In total 588 accessions were found to be moderately resistant with scores 4 and 5 in the UBN screening. Among these lines, the landraces/traditional cultivars together with the accessions of unknown status are also presented.

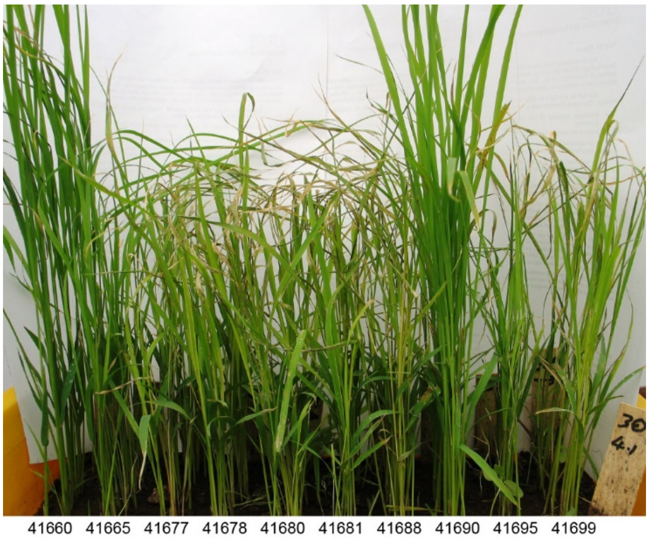

Supplementary figure 4. Isolate specific screening under controlled conditions
Genotypes that were resistant in the uniform blast nursery (scores 0-3) were screened with 5 different rice blast isolates. Tested accessions exhibited varied degrees of resistance reactions against different isolates used. Sample picture of genotypes from India with varying patterns of disease reaction against isolate JMB8401 is presented. The respective IRGC numbers for presented accessions are mentioned.

A

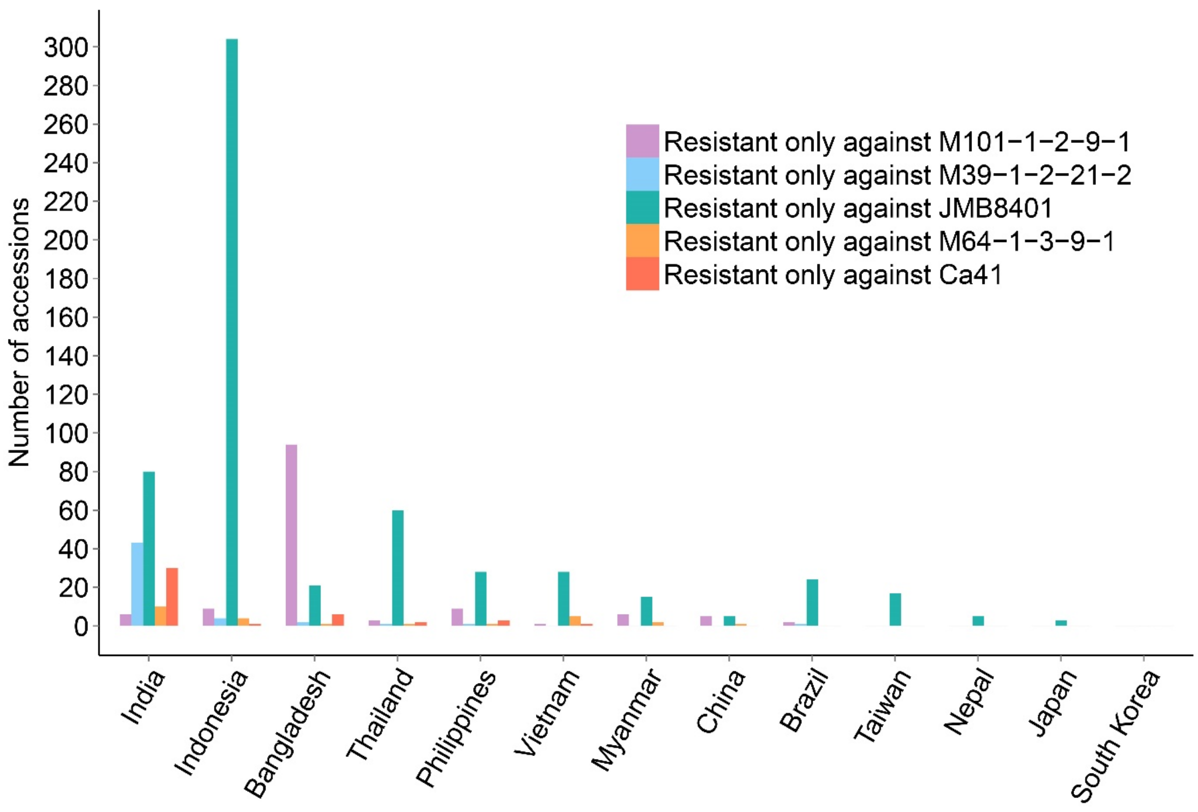

B

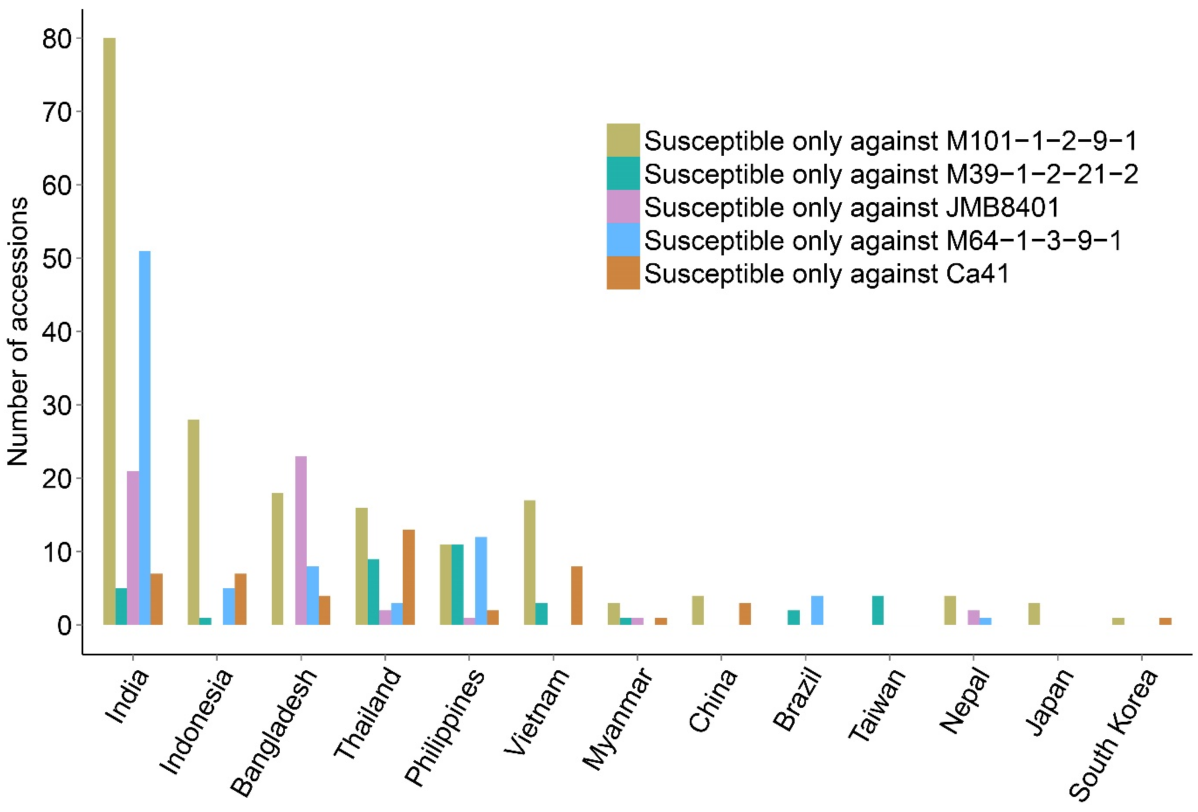

Supplementary figure 5. Accessions specifically resistant or susceptible against each of the five isolates used in the study

The accessions resistant in the uniform blast nursery exhibited varied degree of resistance patterns when tested against individual isolates. Many accessions were resistant against 2 or more isolates. (A) The accessions resistant only to one of the five respective isolates are presented. (B) The accessions susceptible only to one of the five respective isolates are presented.
